# Video-assisted thoracoscopic surgery is a safe and effective method to treat intrathoracic unicentric Castleman’s disease

**DOI:** 10.1186/s12893-020-00789-6

**Published:** 2020-06-10

**Authors:** Yan-qing Wang, Shan-qing Li, Feng Guo

**Affiliations:** Department of Thoracic Surgery, Peking Union Medical College Hospital, Chinese Academy of Medical Sciences & Peking Union Medical College, 100730 No 1 ShuaiFuYuan Street, Dong Cheng District, Beijing, People’s Republic of China

**Keywords:** Castleman’s disease, Video-assisted thoracoscopic surgery, Treatment

## Abstract

**Background:**

Castleman’s disease (CD) is a rare non-clonal lymphadenopathy. Application of video-assisted thoracoscopic surgery (VATs) in intrathoracic unicentric Castleman’s disease (UCD) is rarely reported. This study is aimed to clarify the role of VATs for diagnosis and treatment in intrathoracic UCD.

**Methods:**

The authors reviewed and identified patients who had received a histologic diagnosis of CD through VATs at our hospital from January2010 to June 2018. Clinical and radiologic variables, histopathology, type of approach, complications, and long-term effect were analyzed to evaluate the safety and efficacy of VATs.

**Results:**

A total of 10 patients were included in this study, with 8 hyaline vascular type and 2 plasma cell type. The mean maximum diameter of the lesions was 4.66 cm. Nine cases underwent complete surgical excision by VATs, and 1 case was converted to thoracotomy. All patients had no postoperative complications. With a median follow-up of 5 years (range: 1–9 years), no tumor recurrence was found in 9 patients receiving complete tumor resection, and 1 patient with incomplete tumor resection remained symptom free without clinical or radiographic progression.

**Conclusions:**

VATs is an alternative, minimally invasive technique for the diagnosis and treatment in patients with intrathoracic UCD.

## Background

Castleman’s disease (CD) is a rare non-clonal lymphadenopathy that was first named in 1956 by Castleman et al. [1, 2] Three histopathologic subtypes have been identified, including hyaline vascular, plasma cell, as well as mixed variant [3]. Castleman’s disease can be divided into unicentric type (UCD) and multicentric type (MCD) on the basis of the distribution of enlarged lymph nodes and organ involvement. Hyaline vascular type is more common in UCD but rare in MCD. The vast majority of UCD is mainly located in the chest, and can occur in posterior mediastinal [4], cardiophrenic angle [5], and chest wall [6]. Due to the lack of specificity of enhanced computed tomography, CD was often misdiagnosed as thymoma, lymphoma, hemangiopericytoma, sarcoma, chest wall tumors, neural crest derived neoplasms, bronchial tumor, or pericardial cyst [3, 7]. The diagnosis of Castleman’s disease can only be made by histopathological confirmation.

Surgery plays a critical role in the diagnosis and management of CD since it provides tissue-based diagnosis and greatest chance for cure [8]. It has been considered as the standard therapy for UCD, which was usually performed via conventional thoracotomy [9, 10]. At present, video-assisted thoracoscopic surgery (VATs) is becoming increasingly popular in the thoracic area, which had been proven to be a safe and effective treatment for many thoracic diseases, including mediastinal masses, lung cancer, and pleural diseases [11, 12]. Compared with traditional thoracotomy, VATs provides safer, less invasive resection with shortened hospital stays and faster recovery [13]. However, as far as we know, limited evidence is available about the application VATs in UCD with few studies reporting long-term effects [14–17].

Therefore, here we presented a retrospective analysis by reviewing the CD cases which were diagnosed and treated by VATs in our institution to investigate the role of VATs for the diagnosis and treatment of intrathoracic UCD.

## Methods

### Patients and study design

Consecutive patients with intrathoracic UCD treated initially by VATs between January 2010 and June 2018 at the Department of Thoracic Surgery, Peking Union Medical Hospital (Beijing, China) were enrolled and retrospectively analyzed in this study. Eligible patients had pathologically confirmed CD disease with unknown cause intrathoracic mass requiring surgical exploration.

All patients were subjected to full history taking, collection of clinical manifestations, and laboratory investigations. Thoracic enhancement CT was done in all patients before surgery to record morphologic characteristics and enhancement characteristics of the tumor. Enhancement was defined as low, moderate, and high degree with a cutoff value of less than or equal to 30HU, greater than 30HU but less than 60HU, and greater than 60HU, respectively. All the surgery was conducted by senior attending surgeon, and the pathological examination was confirmed by two experienced pathologists.

The study was approved by the Ethical Committee of Peking Union Medical Hospital, and patient consent was waivered by the local requirement. Written informed consent for image publication was obtained from the patient, and all patient data were anonymized in this study.

### Surgery procedure and follow-up

Under general anesthesia, all procedures were performed under single-lung ventilation with double-lumen endotracheal intubation, which allows collapse of the lung on the operated side. Patients were placed in the lateral position. The trocar for the thoracoscope was positioned at the midaxillary line in the sixth or seventh intercostal space. Finger dissection was used in cases of adhesions of the lung to parietal pleura. Instruments for endoscopic surgery included a rigid thoracoscope, a camera, and reusable instruments. A 30-degree video thoracoscopic camera was inserted through the port. Other trocars were inserted under thoracoscopic visualization. Trocar number and placement varied according to the location of the mass. Most cases required one additional working port other than the camera port. Ports for the placement of operating instruments ranged from 5 to 12 mm in diameter. The dissections were performed using electrocoagulation instruments. Tumors were separated from adjacent organs using gentle blunt and sharp dissecting techniques. Hemostasis was conducted using electrocautery in areas distant from important organs such as vessels or nerves. To prevent tumor cells from spreading during operation, the removal of a dissected tumor was undertaken with extraction sacs. Working port incisions were extended to facilitate the extraction for large mass that was difficult to remove. The operations were completed by insertion of a chest suction tube for drainage through the initial port.

Surgical findings were recorded including the capsular integrity, the status of blood supply of the tumor, the relationship with other structures in the thoracic cavity, invasion to nearby structures, and whether the lesion was completely removed. R0 corresponds to resection for cure or complete remission without residual tumor, R1 to microscopic residual tumor, and R2 to macroscopic residual tumor.

Follow-up was conducted 1 month after surgery, and then every 6 months. Recurrence was defined as new soft tissue lesions in situ for patients with R0 resection, progression was defined as enlarged lesion when compared with preoperative CT findings for R2 patients, otherwise, it is defined as stable disease.

### Data

The primary endpoint was surgical safety, and the secondary endpoint was long-term effect. The blood loss during surgery, the time of operation, postoperative hospital stays, total postoperative drainage, and surgical complications, and the improvement of patients’ symptoms were recorded.

## Results

### Patient characteristics

**Fig. 1:**
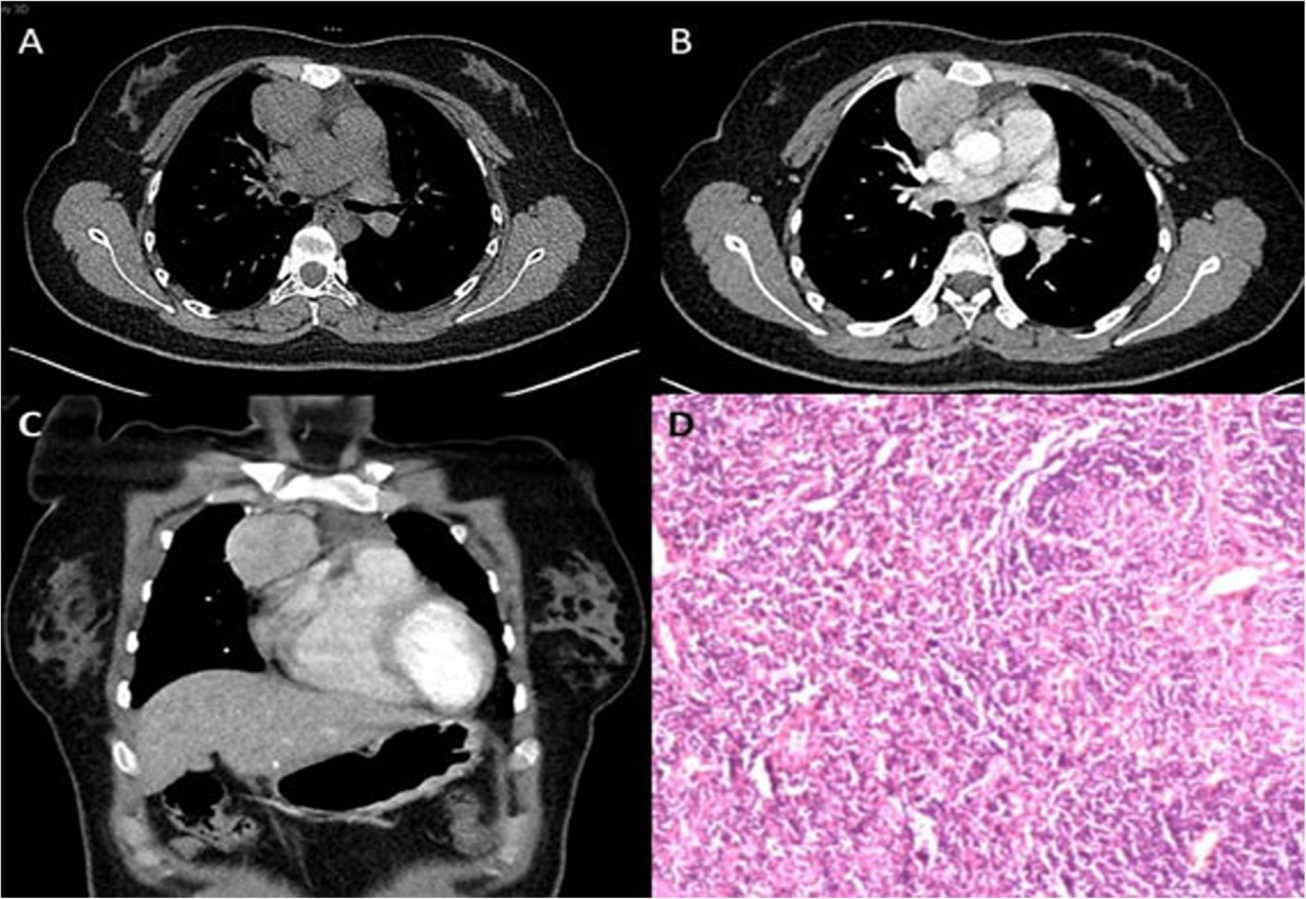
A 21-year-old woman, she has no symptom and was admitted to our hospital because of a mediastinal mass found on a fortuitous CT scan. Contrasted chest CT showed a well-defined and homogeneous enhanced mass in the anterior mediastinum (**a-c**). The mass was whole resected by Video-assisted thoracoscopic surgery and confirmed hyaline vascular type Castleman disease. Histopathologic sections showed that capillary proliferation with perivascular hyalinization in the follicular and interfollicular, with a mixed inflammatory infiltrate of numerous small lymphocytes and plasma cells. (**d**, Hematoxylin and eosin, × 100). Chemotherapy was not suggested. She had been alive without recurrence for 5 years

A total of 10 patients including 7 women and 3 men, with a median age of 32 years (range: 15 to 66 years), were included in the study. Seven patients were detected incidentally by routine physical examination, and the remaining patients presented with chest pain, cough, sputum, and dysphagia. Postoperative pathology diagnosis showed that 8 patients had hyaline vascular CD and others had plasma cell type. A typical case was showed in Fig. 1. All CT findings showed single-lesion mass with 6 located in the anterior mediastinum, and 4 in the middle and posterior mediastinum. The mean tumor size determined by CT was 4.66 cm. Most cases (*n* = 9) had a mass with clear boundary including 3 tumor compressions of surrounding structures, while the remaining 1 showed tumor compression of surrounding esophageal and tracheal bronchus with unclear boundary. High and moderate degree enhancement was seen in 8 and 2 cases on contrast CT, respectively. Table 1 summarizes the clinical characteristics of the patients.

**Table 1:** Clinical characteristics of all patients in the study (*N* = 10)

No	Gender	Age (years)	Disease course (months)	Symptoms	Mass size (cm)	Chest contrast-enhanced CT characteritics	Operative approach	Operative findings
1	female	21	5	None	4.0	Well-defined mass with high-degree enhancement in right anterior mediastinum	Right VATs	Complete capsule with abundant vessels on the surface and tight adhesion to posterior sternum
2	female	66	3	Chest pain	1.9	Well-defined mass with moderate degree enhancement in right cardiophrenic angle	Right VATs	Mass with complete capsule
3	male	52	3	None	3.0	Locating in right paratracheal between the azygous vein arch and SVC with compression of SVC	Right VATs	Complete capsule with clear boundary with SVC
4	female	17	1	None	4.0	Well-defined mass with moderate degree enhancement in right anterior mediastinum	Right VATs	Mass with complete capsule
5	male	24	0.3	None	6.5	Well-defined mass with high-degree enhancement in right paratracheal between the azygous vein arch and SVC	Right VATs	Complete capsule with abundant vessels on the surface
6	female	37	4	Dysphagia	6.4	Mass with high-degree enhancement around the trachea and main bronchus with compression of esophageal and tracheal bronchus	Right VATs #	Incomplete capsule descending to the carina and upward to the subclavian artery with close connection to the trachea
7	male	15	1	Cough, sputum	3.5	Mass with high-degree enhancement in right anterior upper mediastinum with compression tracheal and esophageal	Right VATs	Complete capsule with abundant vessels on the surface and tight adhesion to vagus nerve
8	female	27	1	None	3.3	Well-defined mass with high-degree enhancement and calcification in left anterior upper mediastinum	Left VATs	Complete capsule with abundant vessels on the surface
9	female	40	0.5	None	6	High-degree enhanced mass in left posterior mediastinum, partially extending into the intercostal space	Left VATs	Complete capsule with abundant vessels on the surface
10	female	40	0.3	None	6	Well-defined mass with high-degree enhancement in right anterior mediastinum	Right VATs	Complete capsule with abundant vessels on the surface

*SVC* superior vena cava; # Right VATs converted to thoracotomy, the reason for the conversion to thoracotomy was intraoperative injury of the left main bronchus membrane

### Surgical findings

All patients underwent VATs exploration with a median operation time of 125 min (90–265 min). The mean intraoperative bleeding was 118 ± 88 ml. Most patients had capsuled mass (*n* = 9) with abundant blood vessels (*n* = 6). Dense adhesion between the tumor and the posterior margin of the sternum was seen in 1 patient, while close relation between tumor and the right vagus nerve was seen in another patient. Most patients underwent a complete surgical resection by VATS, while one was converted to thoracotomy due to intraoperative injury of tracheal membrane, which lead to incomplete resection and macroscopic residual tumor. This patient underwent tracheal membrane repair via posterolateral thoracotomy using Prolene (Table 1).

### Treatment outcome

**Fig. 2:**
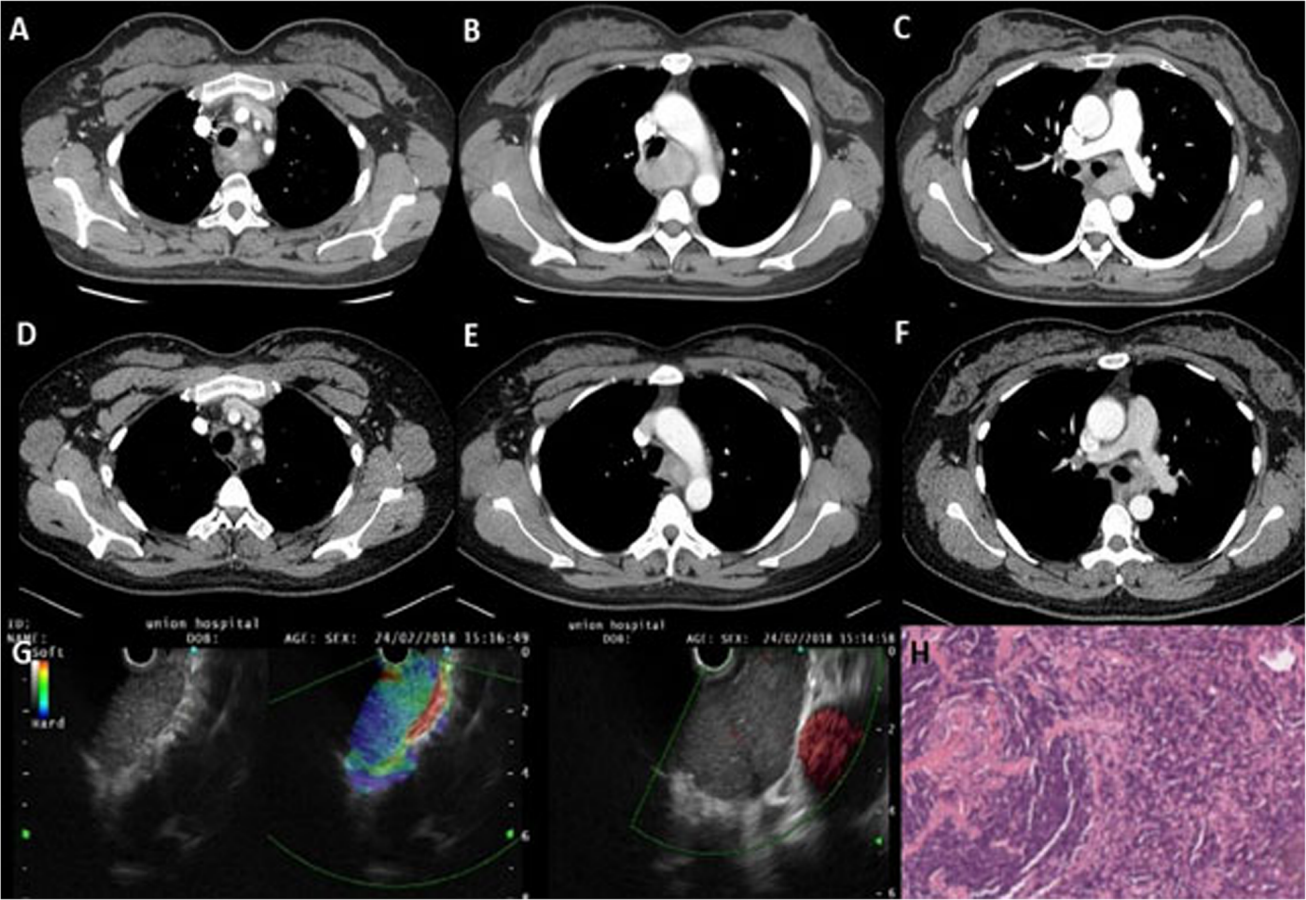
A 37-year-old woman was was admitted to our hospital because of dysphagia for 3 months. Her chest CT showed Irregular soft tissue density, homogeneous enhanced, mass was seen between the trachea and the esophagus. The boundary was not clear, and the trachea, left main bronchus and esophagus were under pressure. **a-c**. Ultrasound gastroscopy showed a hypoechoic mass could be seen in the mediastinum of the esophagus from the incisors 22–26 cm. A clear boundary, irregular edges were seen. The internal echo was still uniform, and no clear necrosis and calcification were seen. It could be seen that small blood vessels pass through the tumor, and the lesions are closely related to the pulmonary blood vessels by using Doppler. **g** This patient converse to thoracotomy because of intraoperative injury of the left main bronchus membrane, and the mass cannot be completely removed. Then she was diagnosed with Castleman disease of hyaline vascular variant in pathology. It was shown that the germinal centers typically form concentric rings, a phenomenon that is knownas “onion skinning”.(H Hematoxylin and eosin, × 200) After 1-year follow-up, chest CT showed no significant progression of the mass. During this period, the patient did not receive any adjuvant treatment (**d-f**)

These patients were discharged from the hospital 5 ± 1 days after the operations. The mean total postoperative thoracic drainage volume was 728 ± 344 ml, and no patient had postoperative complications. By the data cutoff (April 2019), the median follow-up was 5 years (range: 1–9 years). Of the 9 patients following definitive surgical resection, all remain asymptomatic and free of disease at last follow-up. The patient treated with partial resection demonstrated a stable disease with a significant decrease in tumor size (Fig. 2). No patient received postoperative chemotherapy or radiotherapy as adjuvant therapy.

## Discussions

This report reviewed the clinical characteristics, treatment, and outcomes of 10 patients with intrathoracic unicentric Castleman’s disease, indicating that VATs is an acceptable procedure for the treatment and is associated with favorable outcome, fewer complications, and a decreased length of hospital stay. All patients were free of postoperative complications and discharged on schedule. With a five-year follow-up, complete resection of unicentric disease provided a recurrence-free survival for all patients, regardless of histologic subtype, and progression-free survival and decreased tumor size was seen for the patient treated with partial resection.

CD is a rare disease with an unknown cause and characterized by lymph node hyperplasia pathologically. Preoperative diagnosis of intrathoracic UCD is very difficult due to nonspecific clinical manifestations and imaging findings [18–20]. Therefore, the definitive diagnosis of UCD is established histologically by biopsy. Ultrasound-guided endoscopic fine needle aspiration and percutaneous transthoracic puncture biopsy are less invasive and easier to operate. However, aforementioned techniques are associated with potential damage to important structures such as large blood vessels or airways for biopsy in tumors located in the anterior mediastinum or mediastinum. In addition, specimens obtained with these techniques may not provide sufficient material for accurate diagnosis. VATs allows adequate exposure of thoracic cavity and the mass can be either sampled or totally removed, which provides a safe and effective diagnostic procedure for sampling of unicentric CD [16].

Ten patients with UCD treated by VATs in our center were summarized in the present study. Most of them are found by unintentional physical examinations, and a few had symptoms due to local compression by tumor. Non-specific CT findings were seen in most patients, including moderate- to high-degree enhancement of the mass and unclear boundary with surrounding structure, which cannot be distinguished from other thoracic diseases, such as thymoma, lymphoma and malignance of the lung. Ultimately, definitive pathological diagnosis is required by using surgically resected specimen. Intraoperative blood loss was controllable without postoperative complications, indicating VATs is a safe and effective technique for UCDs diagnosis.

Surgical resection has been proven to be an ideal treatment for UCD [20–24]. A systematic review and meta-analysis by Talat et al. summarized data of 404 CD patients from 239 articles between 1954 and 2009 [25]. Of the 278 patients with UCD, 249 underwent surgery, 13 underwent resection combined with immunosuppressive therapy, and 16 received only immunosuppressive therapy. Only 13 patients died from disease-related causes after a ten-year follow-up. These findings demonstrated that surgical resection provide survival benefits in UCD patients and should be considered as the gold standard for treatment.

Open surgery is the traditional form for CD patients since CD lesions are hypervascular [26] and often adhere closely to surrounding tissues or important structures, which needs careful surgical approaching, especially in the mediastinum [27]. However, traditional thoracotomy is traumatic with an unclear visual field as well as prolonged time for operation and postoperative recovery. To date, a limited number of studies have reported the use of VATs for treating CD, and the majority of them were case reports. Table 2 summarized the literatures that reported VATS-treated patients with unicentric Castleman’s disease. In our study, patients’ mass was about 5 cm in diameter with close relation to the surrounding structures. All were explored using VATs which was associated with controllable operation time, less blood loss during operation, shortened postoperative hospital stay. Nine patients achieved complete resection without recurrence after 5-yeay follow-up.

**Table 2:** Video-assisted thoracoscopic surgery treatments and outcomes of patients with unicentric Castleman’s disease in the literature

Reference year	Pathology (sample size)	Mass location	Intervention	Surgical margin	Surgery complication	Outcomes (follow-up period)
Sarana B 2017 [28]	HV (1)	right parahilar tumour	Right VATs converted to thoracotomy^a^+ three-dimensional radiotherapy (cumulative radiation dose of 44 Gy)	R1	No	No recurrence (6-year)
Naomi A 2015 [29]	HV (1)	paravertebral chest wall	Right VATs mass resection	R0	not report	not report
Suh JH 2015 [30]	PC (1)	right mid-superior mediastinum, between the azygous and SVC	Right VATs mediastinal mass resection	R0	No	No recurrence (5-year)
Rawashdeh B 2015 [31]	HV (1)	central portion of left upper lobe	Left VATs left upper lobectomy and mediastinal lymphadenectomy	R0	No	not report
Aoki M 2014 [32]	PC (1)	anterior mediastinum and extended to left pleural cavity	Left VATs, anterior mediastinal adipose tissue, thymus, lesion and all swollen lymph nodes around it resection	R0	No	No recurrence (5-year)
Ishikawa K 2014 [33]	HV (1)	mid-mediastinum, paratracheal between SVC and trachea.	Right VATs mediastinal mass resection	R0	myasthenic crisis	No recurrence (8-year)
Biçakçioğlu P 2014 [34]	HV (16) PC (2) Mix (1)	not report	15 thoracotomy and 3 VATs, 1 mediastinoscopy; biopsies and mass excisions were performed in 2 and 17 cases.	R0	not report	not report
Amano Y 2013 [35]	HV (1)	subcarinal azygoesophageal recess	Embolization of the feeding branches was performed using a gelatin sponge and microcoils; Tumor resection using VATS was performed on the day after the embolization	R0	not report	No recurrence (1-year)
Hideki O 2013 [36]	HV (1)	right lower lobe around the intermediate and basal bronchi	Right VATs right middle-lower lobectomy	R0	No	No recurrence (8-month)
Shohan S 2011 [4]	HV (1)	posterior mediastinal, between azygous vein and esophagus	Right VATs mediastinal mass resection	R0	No	not report
Ichiguchi O 2009 [5]	HV (1)	right cardiophrenic angle	Right VATs mediastinal mass resection	R0	No	not report
Sakairi Y 2009 [37]	HV (2)	right lung hilum	1 thoracoscopic biopsy, excised the right upper lobe, containing the tumor;1 EBUS-TBNA biopsy, excised right middle lobe	R0	No	No recurrence (3/6-year)
Nishii T 2004 [15]	HV (1)	adjacent to the pulmonary artery in the right interlobar fissure	Right VATs mass resection	R0	No	not report
Ko SF 2003 [21]	HV (6) PC (1) Mix (1)	pleura	4 thoracotomies, 2 VATS2; 2 VATS converted to thoracotomy^a^	R0	No	No recurrence (1-16-year)
Seirafi PA 2003 [16]	HV (1)	right paratracheal between the azygous and SVC	Right VATs mediastinal mass resection	R0	No	not report
Iyoda A 2003 [9]	HV (1)	right posterior mediastinal, extended to the tenth intercostal space	Right VATs converted to thoracotomy^a^	R0	No	No recurrence (14-month)

*HV* hyaline vascular type Castleman disease, *PC* plasma cell type Castleman disease, *VATs* video-assisted thoracic surgery, *SVC* superior vena cava; ^a^The reason VATs converted to thoracotomy due to dense adhesions to the adjacent anatomical structures and diffuse bleeding.

For mediastinal and posterior mass with unknown causes, especially for patients with compression symptoms of surrounding structures, such as dyspharyngia, thoracoscopy is only used as an exploration method, and should be actively converted to thoracotomy if thoracoscopic dissection is difficult. In our study, a case with an anterior mediastinal mass, which is usually well demarcated and closely related to the surrounding tissues, especially to vagus nerve, phrenic nerve, and superior vena cava, underwent complete resection by thoracoscopic blunt dissection. It should be noted that intraoperative hemostasis is needed to keep a good visual field during operation. Similar to previous findings by Sarana B [28], Ko SF [21], and Iyoda A et al. [9], we also reported a case with a mass tightly adhered to the trachea and the left pulmonary artery. Membrane of trachea was damaged during operation, and conversion to thoracotomy was required. After the intraoperative repair of the damaged tracheal membrane, the tumor could not be completely removed. The patient underwent chest enhancement CT at 1-year follow-up, and the tumor still existed. Despite failure to complete resection, significant reduction in tumor burden and improved symptoms of dysphagia was seen after surgery (Fig. 2). Therefore, together with published articles, our experience found that tight adhesion of mass to the surrounding structures and bleeding are the main causes of conversion from VATs to thoracotomy. In cases with bleeding risk or unclear relationship with surrounding tissues, VATs can be used for exploration, and timely conversion and repair are required to avoid serious consequences. In addition, our results also suggest that despite failure to completely removal of the mass, acceptable long-term efficacy and symptom improvement could be achieved by partial removal for patients with clinical symptoms.

This retrospective study had several limitations: first, we only enrolled cases accepted VATs, which could result in selection bias. Second, the sample size is small due to the rarity of CD, and the follow-up time was shorter in some cases. The role of VATs needs to be evaluated in prospective studies with larger population and longer follow-up.

## Conclusions

Our study demonstrated VATs is an alternative, minimally invasive technique for the diagnosis and treatment of UCD patients with thoracic mass. Complete resection was performed successfully in patients with well-demarcated masses. Incomplete resection can also achieve satisfying therapeutic effect when the mass was difficult to remove.

## Data Availability

The datasets generated and/or analysed during the current study are not publicly available due to protecting individual patient privacy but are available from the corresponding author on reasonable request.

## Abbreviations

CD: Castleman’s disease
VATs: Video-assisted thoracoscopic surgery
UCD: Unicentric Castleman’s disease
MCD: Multicentric Castleman’s disease
SVC: Superior vena cava
